# Supplementary material for: Paternal methamphetamine exposure induces higher sensitivity to methamphetamine in male offspring through driving ADRB1 on CaMKII-positive neurons in mPFC
Source: Transl Psychiatry. 2023 Oct 19;13:324. doi: 10.1038/s41398-023-02624-x (PMC10587075; doi:10.1038/s41398-023-02624-x)
Supplement: Supplementary file 1 — Supplementary Data [file 41398_2023_2624_MOESM1_ESM.pdf]

Supplementary Data

Supplementary Figure Legends

Figure S1. METH-induced CPP in female F1. a, Experimental design and timeline. **b**, CPP score by female F1. **c**, ΔCPP score by female F1. **d**, Traveled traces in CPP apparatus by female F1. SAL-sired female F1, saline-sired female F1 mice; METH-sired female F1, methamphetamine-sired female F1 mice. N.S., $P > 0.05$ vs Pre-test CPP score or SAL-sired female F1.

Figure S2. The mPFC activity in male F1. a, The number of c-Fos-positive neurons. Scale bar: 500 μm /100 μm. **b**, Density of dendritic spine. Scale bar: 50 μm /10 μm. Cg1, cingulate cortex; PrL, prelimbic cortex; IL, infralimbic cortex. SAL-sired male F1, saline-sired male F1 mice; METH-sired male F1, methamphetamine-sired male F1 mice. *, $P < 0.05$, **, $P < 0.01$, vs SAL-sired male F1.

Figure S3. Effects of sd-METH administration on mPFC activity of naïve mice. a, Experimental design and timeline. **b**, The mRNA levels of *c-Fos* and *Adrb1* in mPFC. **c**, The levels protein of c-Fos and ADRB1 in mPFC. S, saline-injected mice. M, METH-injected mice. N.S., $P > 0.05$ vs S mice.

Figure S4. Effects of betaxolol on METH-preferred behavior in SAL-sired male F1. a, Representative images of cannula location in mPFC. **b**, Timeline of Experimental S1. **c**, CPP score. **d**, ΔCPP score. **e**, Traveled traces in the CPP apparatus by SAL-sired male F1. **f**, Timeline of Experimental S2. **g**, CPP score. **h**, ΔCPP score. **i**, Traveled traces in the CPP apparatus by SAL-sired male F1. F1-SAL-Ts-Vcl, vehicle bilaterally into the mPFC of SAL-sired male F1 at 15 min prior to CPP test; F1-SAL-Ts-Bxol, betaxolol bilaterally into the mPFC of

SAL-sired male F1 at 15 min prior to CPP test; F1-SAL-Tr-Vcl, vehicle bilaterally into the mPFC of SAL-sired male F1 during CPP training; F1-SAL-Tr-Bxol, betaxolol bilaterally into the mPFC of SAL-sired male F1 during CPP training. N.S., $P > 0.05$. ##, $P < 0.01$ vs Pre-test CPP score. **, $P < 0.01$ vs F1-SAL-Ts-Vcl or F1-SAL-Tr-Vcl.

Figure S1

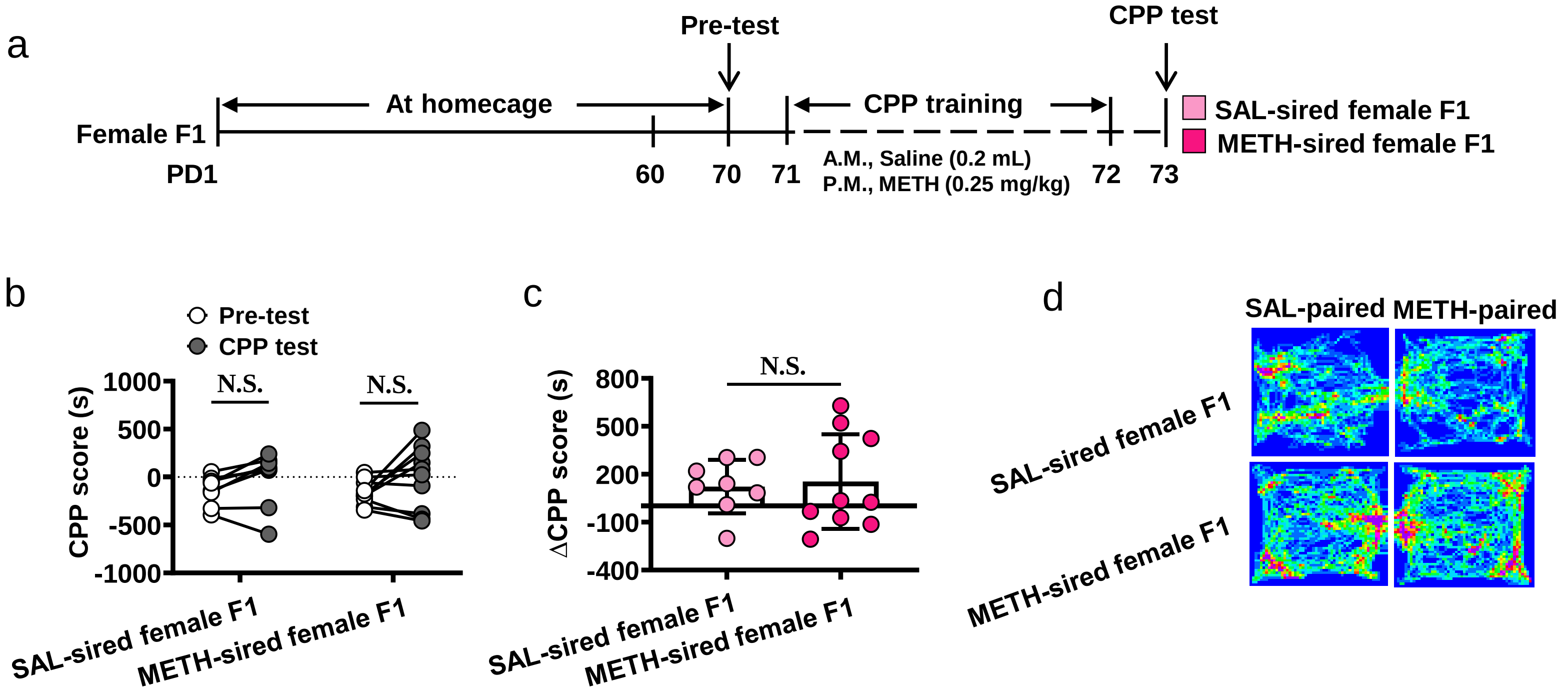
a
Pre-test
CPP test
At homecage
CPP training
Female F1
PD1
60
70
71
A.M., Saline (0.2 mL)
P.M., METH (0.25 mg/kg)
72
73
SAL-sired female F1
METH-sired female F1
b
Pre-test
CPP test
N.S.
N.S.
CPP score (s)
1000
500
0
-500
-1000
SAL-sired female F1
METH-sired female F1
c
N.S.
ΔCPP score (s)
800
500
200
-100
-400
SAL-sired female F1
METH-sired female F1
d
SAL-paired
METH-paired
SAL-sired female F1
METH-sired female F1

Figure S2

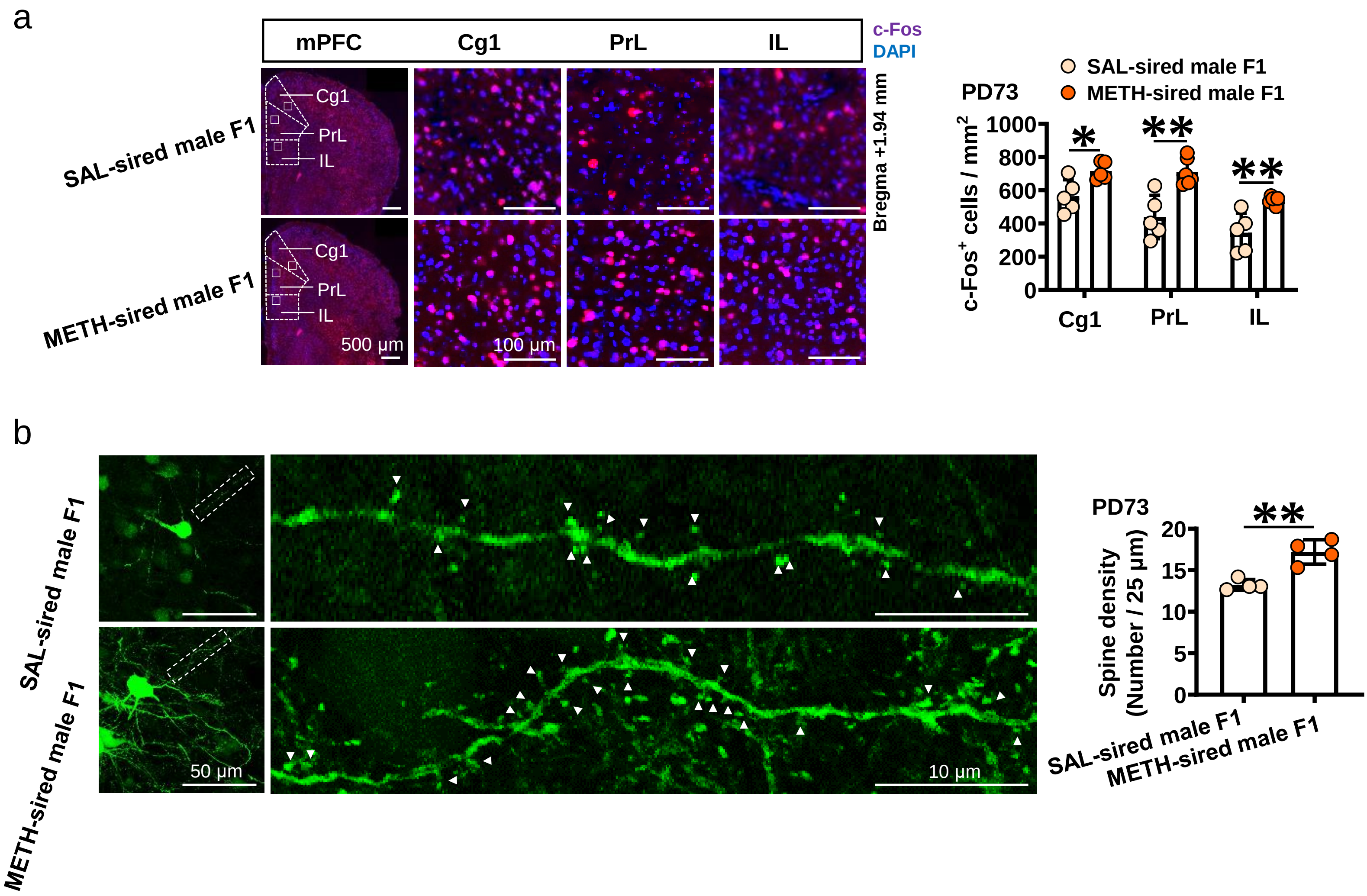
a
mPFC
Cg1
PrL
IL
c-Fos
DAPI
SAL-sired male F1
METH-sired male F1
Cg1
PrL
IL
500 μm
100 μm
Bregma +1.94 mm
PD73
SAL-sired male F1
METH-sired male F1
c-Fos+ cells / mm2
1000
800
600
400
200
0
*
**
**
Cg1
PrL
IL
b
SAL-sired male F1
METH-sired male F1
50 μm
10 μm
PD73
Spine density (Number / 25 μm)
20
15
10
5
0
**
SAL-sired male F1
METH-sired male F1

Figure S3

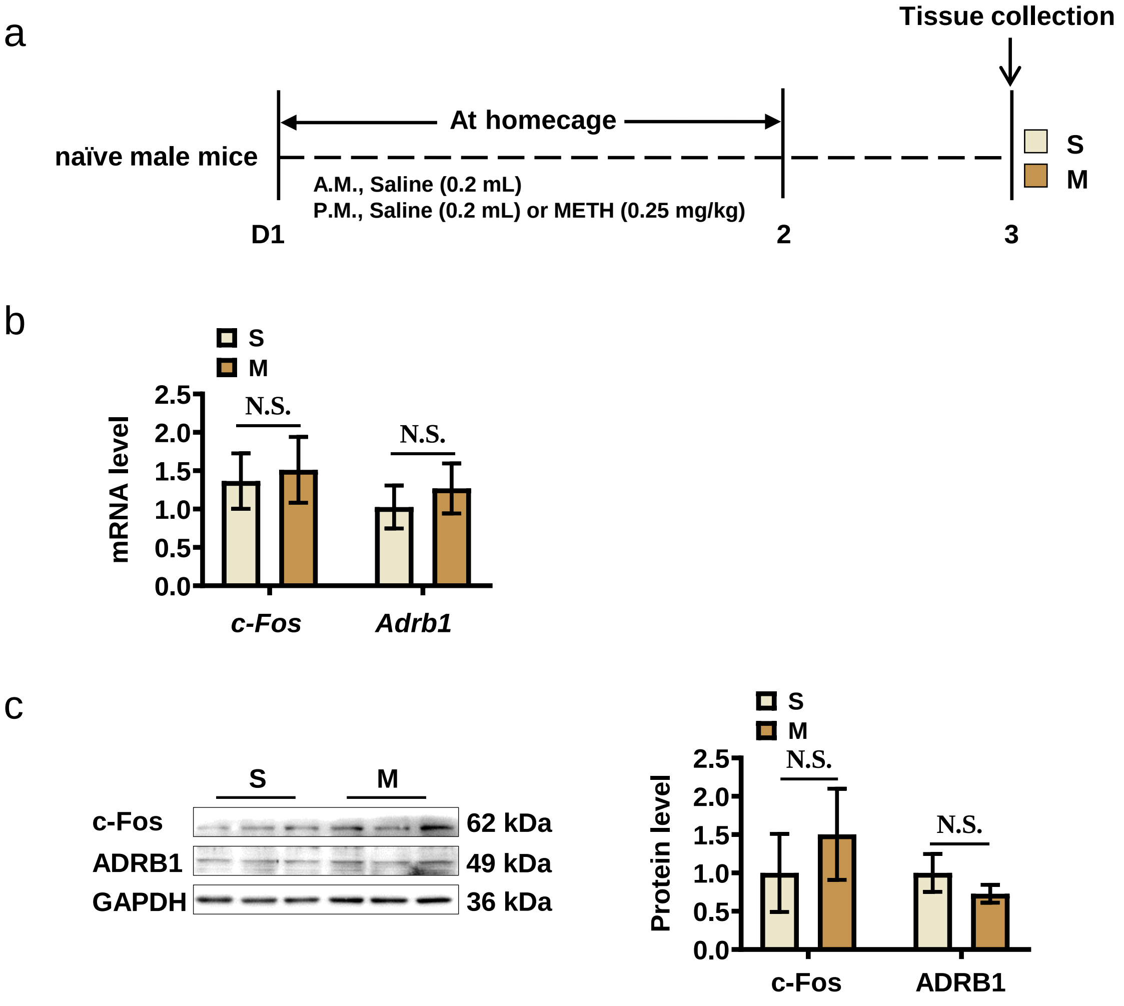
a
Tissue collection
naïve male mice
At homecage
A.M., Saline (0.2 mL)
P.M., Saline (0.2 mL) or METH (0.25 mg/kg)
D1
2
3
S
M
b
S
M
mRNA level
2.5
2.0
1.5
1.0
0.5
0.0
N.S.
N.S.
c-Fos
Adrb1
c
S
M
c-Fos
ADRB1
GAPDH
62 kDa
49 kDa
36 kDa
S
M
Protein level
2.5
2.0
1.5
1.0
0.5
0.0
N.S.
N.S.
c-Fos
ADRB1

Figure S4

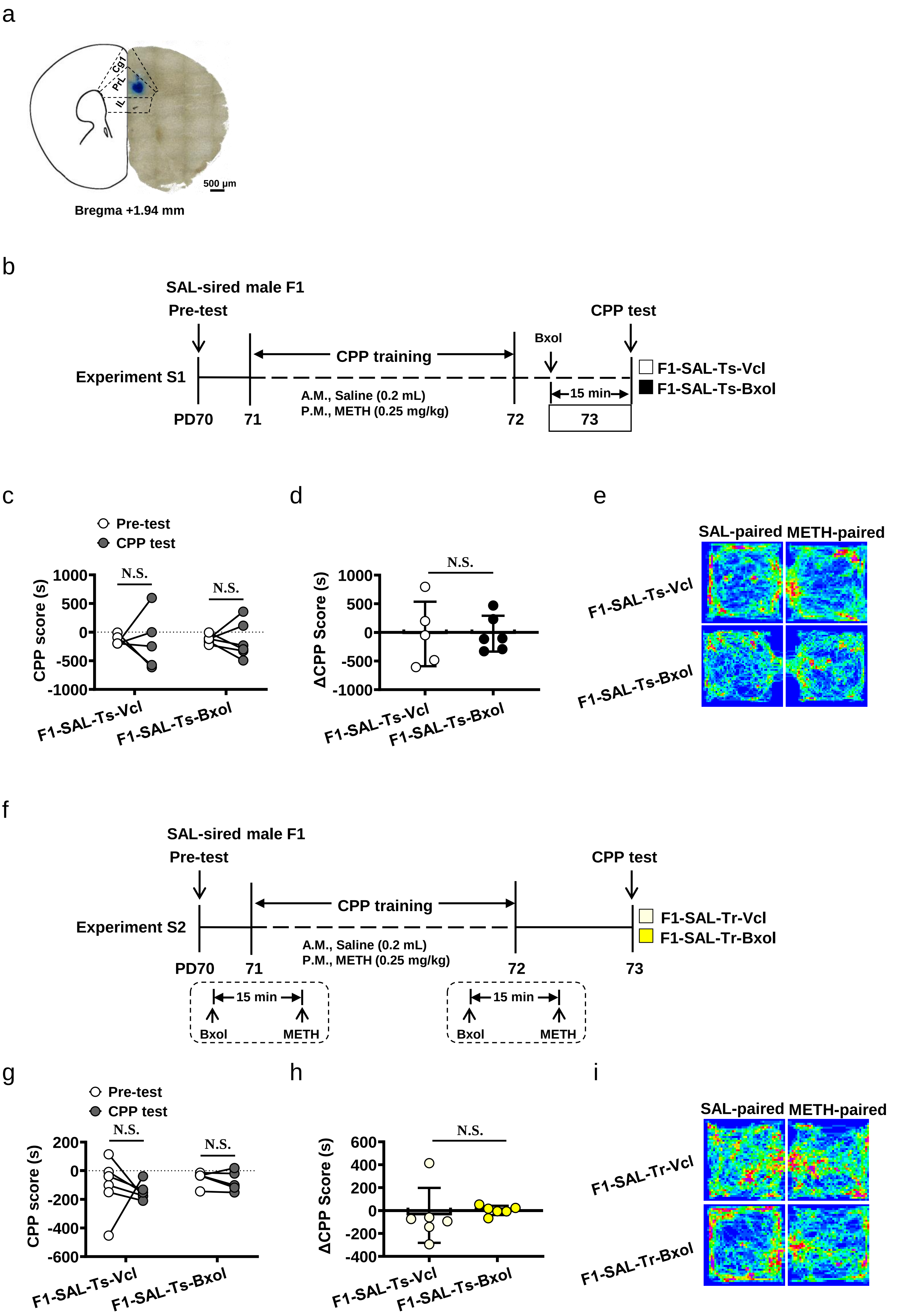
a
Cg1
PrL
IL
500 μm
Bregma +1.94 mm
b
SAL-sired male F1
Pre-test
CPP test
Bxol
CPP training
Experiment S1
F1-SAL-Ts-Vcl
F1-SAL-Ts-Bxol
15 min
A.M., Saline (0.2 mL)
P.M., METH (0.25 mg/kg)
PD70
71
72
73
c
Pre-test
CPP test
N.S.
N.S.
CPP score (s)
1000
500
0
-500
-1000
F1-SAL-Ts-Vcl
F1-SAL-Ts-Bxol
d
N.S.
ΔCPP Score (s)
1000
500
0
-500
-1000
F1-SAL-Ts-Vcl
F1-SAL-Ts-Bxol
e
SAL-paired
METH-paired
F1-SAL-Ts-Vcl
F1-SAL-Ts-Bxol
f
SAL-sired male F1
Pre-test
CPP test
CPP training
Experiment S2
F1-SAL-Tr-Vcl
F1-SAL-Tr-Bxol
A.M., Saline (0.2 mL)
P.M., METH (0.25 mg/kg)
PD70
71
72
73
15 min
Bxol
METH
15 min
Bxol
METH
g
Pre-test
CPP test
N.S.
N.S.
CPP score (s)
200
0
-200
-400
-600
F1-SAL-Ts-Vcl
F1-SAL-Ts-Bxol
h
N.S.
ΔCPP Score (s)
600
400
200
0
-200
-400
F1-SAL-Ts-Vcl
F1-SAL-Ts-Bxol
i
SAL-paired
METH-paired
F1-SAL-Tr-Vcl
F1-SAL-Tr-Bxol
